# Development of the α-IGZO/Ag/α-IGZO Triple-Layer Structure Films for the Application of Transparent Electrode

**DOI:** 10.3390/ma10030226

**Published:** 2017-02-24

**Authors:** Kun-Neng Chen, Cheng-Fu Yang, Chia-Ching Wu, Yu-Hsin Chen

**Affiliations:** 1 Department of Electrical Engineering, Kun-Shan University, Tainan 710, Taiwan; knchen@mail.ksu.edu.tw; 2 Department of Chemical and Materials Engineering, National University of Kaohsiung, Kaohsiung 811, Taiwan; cfyang@nuk.edu.tw (C.-F.Y.); j1040404@gmail.com (Y.-H.C.); 3 Department of Electronic Engineering, Kao Yuan University, Kaohsiung 82151, Taiwan

**Keywords:** amorphous IGZO, Ag, triple-layer structures, transmittance, electrical conductivity

## Abstract

We investigated the structural, optical, and electrical properties of amorphous IGZO/silver/amorphous IGZO (α-IGZO/Ag/α-IGZO) triple-layer structures that were deposited at room temperature on Eagle XG glass and flexible polyethylene terephthalate substrates through the sputtering method. Thin Ag layers with different thicknesses were inserted between two IGZO layers to form a triple-layer structure. Ag was used because of its lower absorption and resistivity. Field emission scanning electron microscopy measurements of the triple-layer structures revealed that the thicknesses of the Ag layers ranged from 13 to 41 nm. The thickness of the Ag layer had a large effect on the electrical and optical properties of the electrodes. The optimum thickness of the Ag metal thin film could be evaluated according to the optical transmittance, electrical conductivity, and figure of merit of the electrode. This study demonstrates that the α-IGZO/Ag/α-IGZO triple-layer transparent electrode can be fabricated with low sheet resistance (4.2 Ω/□) and high optical transmittance (88.1%) at room temperature without postannealing processing on the deposited thin films.

## 1. Introduction

Amorphous transparent conductive oxide (a-TCO) is promising for emerging large-area optoelectronic applications because of capability of large-area, uniform deposition at low temperatures such as room temperature (RT) [1,2,3]. The ease of manufacturability at room temperature makes a-TCO materials very attractive, because at such a low temperature, the a-TCO materials show smooth surfaces, which are advantageous for process integration. Also, the problem of having grain boundaries can also be avoided. The a-TCO materials have no long-range atomic order and they are not only transparent in the visible region but also reveal highly electrical conduction [4]. In addition, the a-TCO have unique carrier transport properties, for example with Hall mobility larger than 15 cm^2^·(V·s)^−1^ [5,6]. Amorphous indium gallium zinc oxide (α-IGZO) is a base N-type semiconductor and satisfies the application of various requirements. For example, α-IGZO has a very high transmittance in the visible range and a large electron mobility of 11.0~163.4 cm^2^/V-s [7]. For that, so many researchers have intensively studied the investigation of high quality α-IGZO films [7].

It is well known that metal film shows a good conductivity but a low transparency in the visible range. The structure, which combines TCO with a thin metal layer to achieve a stacked layer with low resistivity and high transparency, has been developed. The TCO/metal/TCO triple-layer structure, consisting of two outer oxide layers and a middle layer of thin metal film, has been intensively investigated for various applications [8,9,10]. However, to achieve metallic conductivity with TCO without significant loss in transmittance, various thin metals like Ag, Cu, Au, Pt, and Al had been used as the embedded metal layer of the TCO/metal/TCO triple-layer structure [11,12,13]. Especially, the use of a thin Ag layer 10–15 nm inserted between two TCO layers to form a triple-layer (or called as sandwich) has been widely studied. The thickness of the thin metal layer significantly influences the optical and electrical properties of the multilayer stacks [14,15]. Compared with single-layer TCO thin films, the TCO/metal/TCO triple-layer structure can effectively suppress the reflection from the metal layer in the visible range and yield better electrical conductivity. The conventional amorphous indium tin oxide (ITO) films grown on polymer substrates have the problems of high sheet resistance, low transition temperature from amorphous to crystalline phase, and rapid deterioration by substrate bending [16], therefore, to solve these problems become important. The TCO/metal/TCO triple-layer electrodes have those properties, and they can be investigated to substitute amorphous ITO electrodes as a flexible electrode for flexible organic light-emitting diodes (OLEDs) and organic photovoltaic devices (OPVs). So far, various ITO/metal/ITO triple-layer structures have been reported, for example, IZTO (indium zinc tin oxide)/Ag/IZTO [16], ITO/Ag/ITO [17,18], GZO/Ag/GZO [19], AZO/Ag/AZO [20], ZnO/Cu/ZnO [21], AZO/Au/AZO [22], and graphene/ITO [23]. 

In previous reports, the higher transmittance of a TCO/Ag/TCO electrode than that of a TCO/Au/TCO electrode was explained by the lower absorption of Ag than that of Au in the visible region of 500–800 nm (5% vs. 8%) [24,25,26]. For that reason, in this research we chose Ag as the material of thin metal film to fabricate multi-layer TCO films with the α-IGZO/Ag/α-IGZO triple-layer structures. In this work, we compared the structural, electrical, and optical properties of the α-IGZO/Ag/α-IGZO triple-layer structures were fabricated using radio frequency (RF) magnetron sputtering method at room temperature. The Ag and α-IGZO as the thin metal film and TCO film, respectively, and so far the thickness variation of Ag film on the properties of α-IGZO/Ag/α-IGZO triple-layer structures are not reported. The structural, optical, and electrical properties of α-IGZO/Ag/α-IGZO triple-layer structures were characterized by field emission scanning electron microscopy (FE-SEM), X-ray diffraction (XRD), ultraviolet (UV)-visible spectroscopy, and Hall measurement, respectively. The figure of merit (FOM) was also calculated to characterize the performance of the triple-layer structures. In this study, by inserting a very thin layer of Ag between two layers of amorphous IGZO, we could fabricate a highly flexible, low resistance, and highly transparent IGZO-Ag-IGZO triple-layer electrode on the glass and polyethylene terephthalate (PET) substrates.

## 2. Experimental Procedures

The amorphous In-Ga-Zn-O (α-IGZO) film and the silver (Ag) thin metal film were deposited at room temperature by radio frequency (RF) magnetron sputtering method on Corning Eagle XG glass and PET substrates. High purity raw materials of In_2_O_3_, Ga_2_O_3_, and ZnO with purity higher than 99.9% were weighed according to the formula composition In_2_O_3_-Ga_2_O_3_-2 ZnO (abbreviated as IGZO) in mole ratio and ball-milled with alcohol for 24 h in polyethylene (PE) bottles. After being dried and ground, the mixed IGZO powder was calcined at 1100 °C for 2 h. According to the XRD analysis, the as-calcined powders revealed an IGZO phase [27]. Finally, the calcined IGZO powder was mixed with PVA as binder and uniaxially pressed into a pellet of 5 mm thickness and 54 mm diameter using a steel die. The IGZO pellet was sintered at 1350 °C in air for 6 h to prepare the ceramic target. The sputtering deposition parameters of α-IGZO film and Ag thin metal film were base pressure of 3 × 10^−6^ torr, Ar flow rate of 20 sccm, working pressure of 5 × 10^−2^ torr, and target-substrate distance of 5 cm, and RF power for α-IGZO was 100 W and for Ag was 40 W. Prior to deposition, both the IGZO and Ag targets were pre-sputtered for 5 min to remove surface contaminants. At first, the thickness of the both top and bottom layer of α-IGZO film was set at 40 nm and the thicknesses of Ag thin metal films were 10, 11.5, 13, 14.5, and 16 nm, respectively, to find α-IGZO/Ag/α-IGZO triple-layer structures with better characteristics. After that, the thickness of Ag thin metal film was set at 13 nm, the thicknesses of the both top and bottom layers of α-IGZO film in the α-IGZO/Ag/α-IGZO triple-layer structures were changed from 27 nm to 69 nm, respectively.

The surface microstructure and cross-sectional observation of the deposited α-IGZO/Ag/α-IGZO triple-layer structures were examined by field emission scanning electron microscopy (FE-SEM). Crystalline structures of the α-IGZO/Ag/α-IGZO triple-layer structures were characterized by X-ray diffractometer using CuKα radiation. The optical transmittance spectrum of the α-IGZO/Ag/α-IGZO triple-layer structures were measured using a Hitachi U-3300 UV-Vis spectrophotometer in the 200 to 1000 nm wavelength range. The resistivity (ρ), carrier concentration (n), and mobility (μ) of the α-IGZO/Ag/α-IGZO triple-layer structures were obtained from Hall-effect measurements.

## 3. Discussion

**Figure 1:**
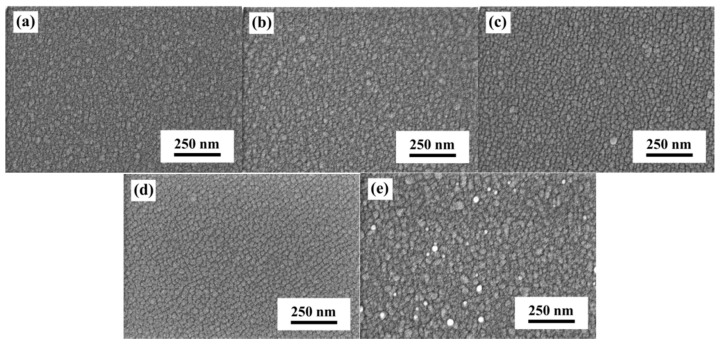
Surface SEM observations of the α-IGZO/Ag/α-IGZO triple-layer structures as a function of thickness of Ag film. (**a**) 10; (**b**) 11.5; (**c**) 13; (**d**) 14.5; and (**e**) 16 nm, respectively.

The surface SEM images of the α-IGZO/Ag/α-IGZO triple-layer structures as a function of thickness of Ag film are shown in Figure 1, and the thickness of the top and bottom α-IGZO film was controlled at 40 nm. The results in Figure 1 indicate that as thickness of Ag film was changed, the surface morphologies of the α-IGZO/Ag/α-IGZO triple-layer structures showed different results. As the thickness of Ag film was increased from 10 nm to 16 nm, the surface morphologies of the α-IGZO/Ag/α-IGZO triple-layer structures exhibited a very smooth surface regardless of thickness of Ag film, and the nano-crystallization grains were clearly observed. The surface morphology of the α-IGZO/Ag/α-IGZO triple-layer structure was smooth as the thickness of Ag film was 10 nm. The particle sizes of nano-crystallization grains increased with the increase of thickness of Ag film. Small grains in the α-IGZO/Ag/α-IGZO triple-layer structure appeared as the Ag thickness grew larger than 11.5 nm. The average particle sizes of nano-crystallization α-IGZO grains can be calculated using the following equation [28]:

*G* = −2.9542 + 1.4427 ln(*N*) (1)

where *G* is the number of grains per unit area at a particular magnification, *N* is the number of grains/mm^2^. As the thicknesses of Ag film was 11.5 nm-, 13 nm-, 14.5 nm-, and 16 nm, the average particle sizes of nano-crystallization grains were about 35.8 nm, 39.6 nm, 43.5 nm, and 54.3 nm, respectively, as shown in Figure 1b–e. The increase of particle size and roughness with the increase of thickness of Ag film in the IGZO/Ag/IGZO triple-layer structure is caused by the transformation of the Ag surface morphology from isolate to a continuous film [29]. 

**Figure 2:**
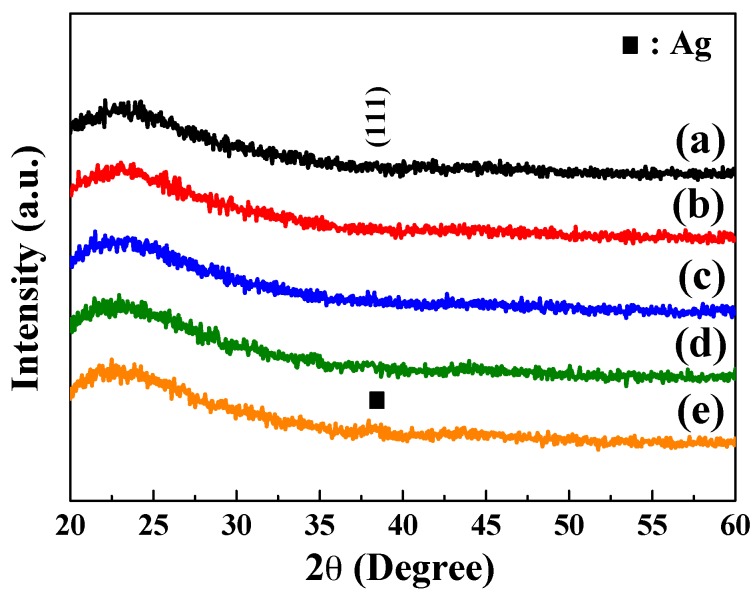
XRD analysis of the α-IGZO/Ag/α-IGZO triple-layer structures as a function of thickness of Ag film. (**a**) 10; (**b**) 11.5; (**c**) 13; (**d**) 14.5; and (**e**) 16 nm, respectively.

As the different sintering temperatures were used, the differently crystalline phases would be formed in the IGZO ceramic targets, and the multi-crystal phases were really observed in the IGZO ceramic targets (not shown here). The crystal characteristics of the α-IGZO/Ag/α-IGZO triple-layer structure as a function of thickness of Ag film were investigated using XRD analysis, and the results are presented in Figure 2. IGZO and Ag (or α-IGZO/Ag/α-IGZO triple-layer) films deposited by the sputtering method in the pure Ar would reveal the amorphous phase rather than the polycrystal phase since no diffraction characteristic peaks were observed in the XRD patterns of the α-IGZO/Ag/α-IGZO triple-layer structures. The broad peak appearing in the range of 2θ = 20°~30° was assigned to the glass substrate or the α-IGZO film with the all α-IGZO/Ag/α-IGZO triple-layer films. The weak peak appearing at 2θ = 38.2° was assigned to the (111) diffraction peak of Ag film with a thickness of 16 nm. Those results demonstrated that all the IGZO films deposited at room temperature exhibit the amorphous phase and the thinner Ag layer embedded in middle region of the α-IGZO/Ag/α-IGZO triple-layer structure. The amorphous natures of the deposited IGZO and Ag films can result from the low temperature of the sputtering process.

**Figure 3:**
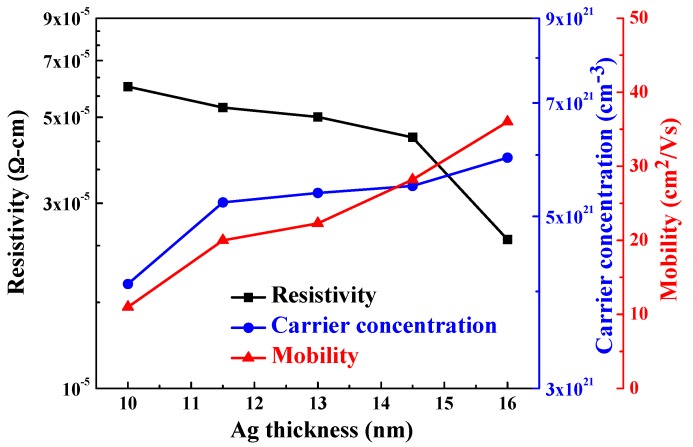
Hall measurement of the α-IGZO/Ag/α-IGZO triple-layer structure as a function of thickness of Ag film, where thickness was changed from 10 nm to 16 nm.

The resistivity (ρ), carrier concentration (n), and mobility (μ) of the α-IGZO/Ag/α-IGZO triple-layer structure as a function of thickness of Ag film were compared in Figure 3, the thickness of the top and bottom α-IGZO film was controlled at 40 nm. The carrier concentration increased from 4.09 × 10^21^ cm^−3^ to 5.99 × 10^21^ cm^−3^ as thickness of Ag film increased from 10 nm to 16 nm. This result caused by the carriers in the Ag layer can be easily injected into α-IGZO layer due to downward band bending by the difference in work functions between Ag layer and α-IGZO in the α-IGZO/Ag/α-IGZO triple-layer structures [30]. In addition, the mobility of the α-IGZO/Ag/α-IGZO triple-layer structure increased from 11 cm^2^/V to 36 cm^2^/V as the thickness of Ag film increased. The reason is largely ascribed to the reduction of scattering at the interface regions between the metal and oxide layers, since the interface scattering is considered the main scattering mechanism in the α-IGZO/Ag/α-IGZO triple-layer structure [31]. In addition, the large mobility of α-IGZO/Ag/α-IGZO triple-layer structure is caused by the α-IGZO as the top and bottom TCO films [6]. The resistivity of the α-IGZO/Ag/α-IGZO triple-layer structure slightly decreased from 5.99 × 10^−5^ to 4.45 × 10^−5^ Ω-cm as the Ag thicknesses increased from 10 nm to 14.5 nm and it rapidly decreased to 2.39 × 10^−5^ Ω-cm as the Ag thickness at 16 nm. The decrease in resistivity is mainly due to the increases of both carrier concentration and mobility with the increase of thickness of Ag film because the resistivity of the α-IGZO/Ag/α-IGZO triple-layer structure is proportional to the reciprocal value of the product of the carrier concentration N and the mobility μ [32]

*Ρ* = 1/(N_e_μ) (2)

The minimum resistivity of the α-IGZO/Ag/α-IGZO triple-layer structure at an Ag thickness of 16 nm is mainly influenced by both the carrier concentration and the carrier mobility being at their maximum [33].

**Figure 4:**
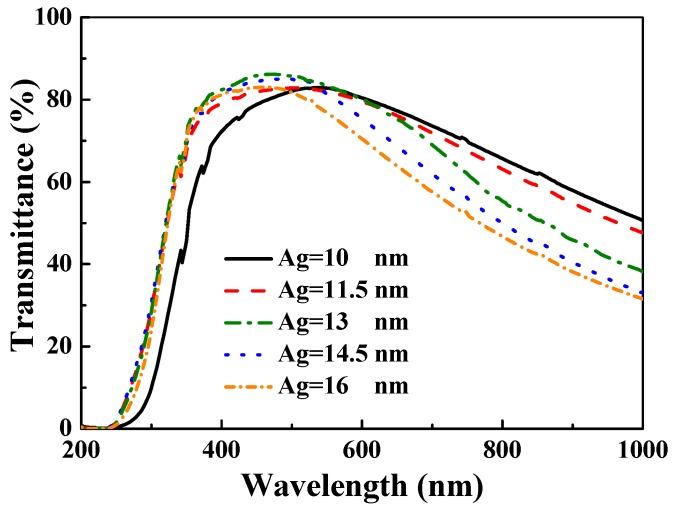
Ultraviolet-visible spectra of the α-IGZO/Ag/α-IGZO triple-layer structure as a function of thickness of Ag film.

The optical transmittance spectra of the α-IGZO/Ag/α-IGZO triple-layer structure as a function of thickness of Ag film are shown in Figure 4, where thickness of IGZO films at the top and lower layers were still controlled at 40 nm. In the Figure 4, all of the samples showed a sharp optical band edge in the UV region, and the optical band edge was also shifted to a shorter light wavelength as the thickness of Ag film increased. The transmittance ratios of the all α-IGZO/Ag/α-IGZO triple-layers were 80%–85% in the visible region, subsequently the transmittance ratios decreased to about 30%–60% in the near infrared region. The transmittance ratio at 480 nm was measured to be 80.6, 82.2, 86.3, 84.9, and 82.8% for the 10, 11.5, 13, 14.5, and 16 nm Ag thicknesses, respectively. The maximum transmittance of 86.3% at 480 nm observed in the α-IGZO/Ag/α-IGZO triple-layer structure with Ag thickness of 13 nm is related to antireflection effects [24]. 

As explained in previous reports, at the beginning of deposition, Ag particles were deposited as separate islands randomly distributed on bottom α-IGZO film. As the deposition time (or the thickness) increased, the Ag islands coalesced to a continuous film and it would work as a reflection film. When an optimum Ag metal film layer is embedded between two oxide layers, the resulting symmetric oxide/metal/oxide (OMO) triple-layer structure can suppress reflections from the Ag metal film layer which results in a high optical transmittance in the visible wavelength region [9,33,34,35]. However, as the thickness of Ag is further increased, the transmittance ratio of OMO triple-layer structure decreases due to the opaqueness of the thicker Ag metal film. In particular, the transmittance of the α-IGZO/Ag/α-IGZO triple-layer structure in the infrared wavelength region significantly decreases with the increase in thickness of Ag film, because the plasma frequency (ω_p_) depends on the carrier concentration of the Ag metal film.

**Figure 5:**
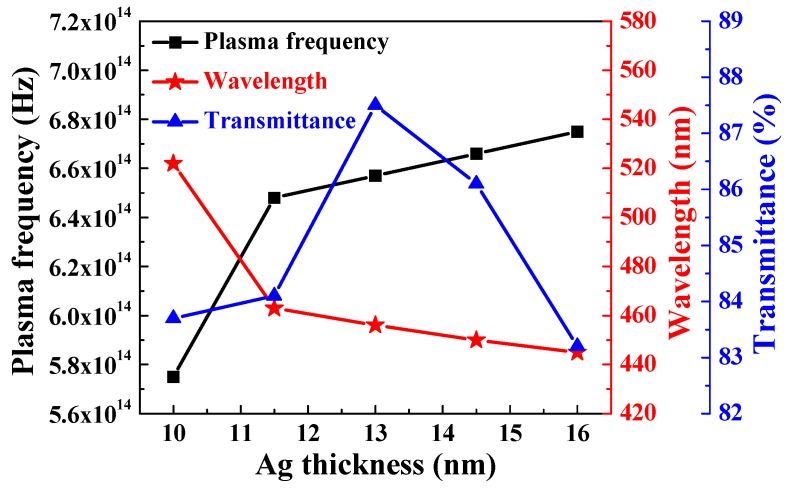
Plasma frequency of the α-IGZO/Ag/α-IGZO triple-layer structure as a function of thickness of Ag film.

As the thickness of Ag film increased from 10 nm to 11.5 nm, the optical band edges in the transmission spectra of the α-IGZO/Ag/α-IGZO triple-layer structure were shifted to a shorter light wavelength and a greater sharpness was noticeable in the curves of the absorption edges. The blue-shift of the maximum transmittance in the α-IGZO/Ag/α-IGZO triple-layer structure was also apparently observed as the thickness of Ag film increased. Just as shown in Figure 3, the carrier concentration of the α-IGZO/Ag/α-IGZO triple-layer structure increased with the increasing thickness of Ag film. Assuming a homogeneous material, a higher carrier concentration would result in a larger plasma frequency. Figure 5 shows plasma frequency of the α-IGZO/Ag/α-IGZO triple-layer structure as a function of thickness of Ag film. Plasma frequency ω_p_ can be explained and calculated using the following equation:

(3) $$\omega_{p}=\sqrt{\frac{N_{e}\times e^{2}}{m\times \varepsilon_{0}}}$$

where N*_e_* is the carrier concentration, e is the electric charge, m is the effective mass of the electron, and ε_0_ is the permittivity of free space [36]. It is found that the plasma frequency of the α-IGZO/Ag/α-IGZO triple-layer structure increases from 5.74 × 10^14^ to 6.75 × 10^14^ Hz and the wavelength (λ) shifts from the near infrared region to the visible region as the function of Ag thickness, as shown in Figure 5.

**Figure 6:**
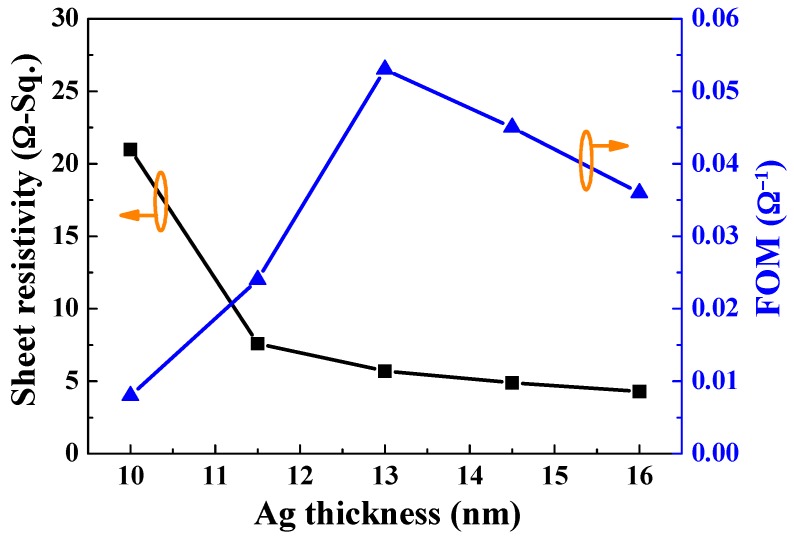
FOM values of the α-IGZO/Ag/α-IGZO triple-layer structure as a function of thickness of Ag film.

Figure 6 shows a calculated FOM (*φ_TC_*) of the α-IGZO/Ag/α-IGZO triple-layer structure as a function of thickness of Ag film. FOM was calculated using Equation (4)

(4) $$\varphi_{TC}=\frac{T_{av}^{10}}{R_{s}}$$

where *T_av_* is the average optical transmittance and *R_s_* is the sheet resistance [37]. *T_av_* can be estimated using Equation (5)

(5) $$T_{av}=\frac{\int v\left(\lambda \right)T\left(\lambda \right)d\left(\lambda \right)}{\int v\left(\lambda \right)d\left(\lambda \right)}$$

where *T*(λ) is the transmittance and *v*(λ) is the photopic luminous efficiency function defining the standard observer for photometry [38]. In the Figure 6, the FOM value first increased with the thickness of Ag film, reached a maximum at 13 nm, and then decreased as the thickness of Ag film further increased. The Ag film with thickness of 13 nm shows a maximum FOM of 0.053 Ω^−1^. The sheet resistance and average optical transmittance were 5.7 Ω/□ and 87.5%, respectively. This result implies the FOM value of the α-IGZO/Ag/α-IGZO triple-layer structure is an important factor to affect the average transmittance rather than to affect the sheet resistance.

**Figure 7:**
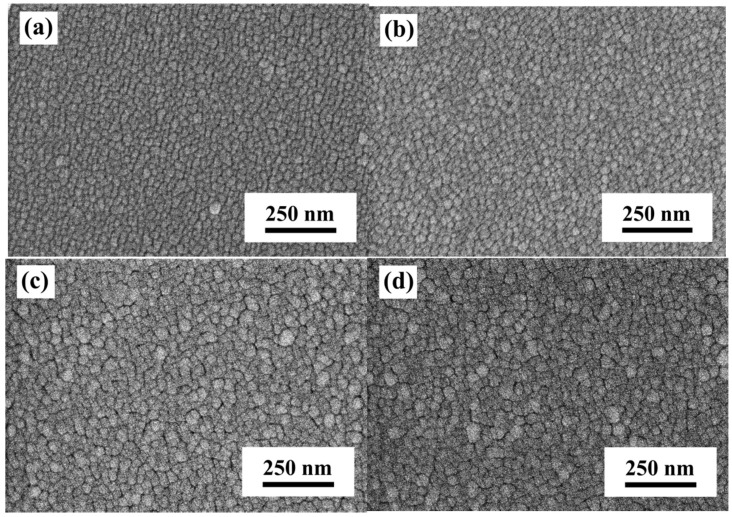
Surface SEM images of the α-IGZO/Ag/α-IGZO triple-layer structure as a function of thickness of IGZO film, where thickness was (**a**) 27; (**b**) 41; (**c**) 55; and (**d**) 69 nm, respectively.

The optimal optical properties of the α-IGZO/Ag/α-IGZO triple-layer structure were obtained as the thickness of Ag film was 13 nm. To further reduce the resistivity and enhance the transmittance of the α-IGZO/Ag/α-IGZO triple-layer structure, the thickness of Ag film was set at 13 nm, the α-IGZO top and bottom films with different thicknesses were investigated on the glass and on PET substrates for possible applications of flexible substrates. Figure 7 shows the surface SEM images of the α-IGZO/Ag/α-IGZO triple-layer structure on glass substrate as a function of thickness of α-IGZO films. Small grains in the α-IGZO/Ag/α-IGZO triple-layer structure appeared as the α-IGZO thickness at 27 nm and the average grain size was about 14.5 nm. The average grain size of the α-IGZO/Ag/α-IGZO triple-layer structure increased with the increase in the thickness of α-IGZO films. As the thicknesses of α-IGZO films were 27, 41, 55, and 69 nm, the average grain sizes of the α-IGZO/Ag/α-IGZO triple-layer structures on glass substrate were about 14.5, 27.3, 49.1, and 58.7 nm, respectively, as shown in Figure 7a–d. The α-IGZO/Ag/α-IGZO triple-layer structure was also deposited on PET substrate. Also, as the thicknesses of α-IGZO films were 27, 41, 55, and 69 nm, the average grain sizes of the α-IGZO/Ag/α-IGZO triple-layer structures were about 12.7, 24.2, 43.8, and 52.2 nm, respectively (not shown here).

**Figure 8:**
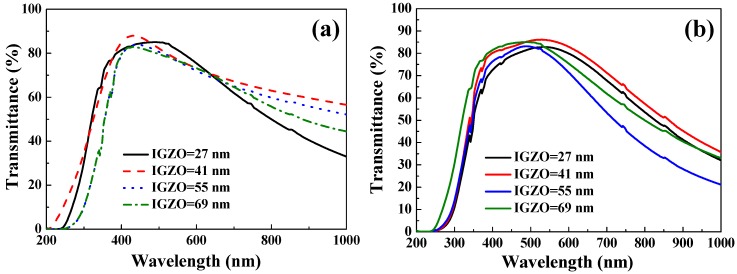
UV-vis spectra of the α-IGZO/Ag/α-IGZO triple-layer structure as a function of thickness of IGZO films. On (**a**) glass substrates; and (**b**) PET substrates.

The transmittance properties obtained from the α-IGZO/Ag/α-IGZO triple-layer structure with Ag film thickness of 13 nm, and as a function of thickness of α-IGZO films, were also measured and the results are shown in Figure 8. As Figure 8a shows, as glass was used as substrate, the maximum transmittance is measured to be 85.6%, 88.1%, 83.9%, and 82.8% as the thickness of α-IGZO films were 27, 41, 55, and 69 nm, respectively. As Figure 8b shows, as flexible PET was used as substrate, the maximum transmittance was measured to be 82.8%, 85.7%, 83.5%, and 84.6% as the thickness of α-IGZO films were the 27, 41, 55, and 69 nm, respectively. Deposition of the bottom and top α-IGZO layers with different thicknesses led to an increase in the optical transmittance ratio of the α-IGZO/Ag/α-IGZO triple-layer structures as the thickness of α-IGZO films was increased to 41 nm. This result indicates that the α-IGZO layers with a thickness of 41 nm are desirable to realize an antireflection effect in the α-IGZO/Ag/α-IGZO triple-layer structure. However, a further increase in the thickness of the α-IGZO films to 55 nm and 69 nm resulted in an apparent decrease of the transmittance ratio. From these results, it is obvious that the optimum thickness of α-IGZO films in the inverted α-IGZO/Ag/α-IGZO triple-layer structure is effective to improve the optical properties in the visible region.

**Figure 9:**
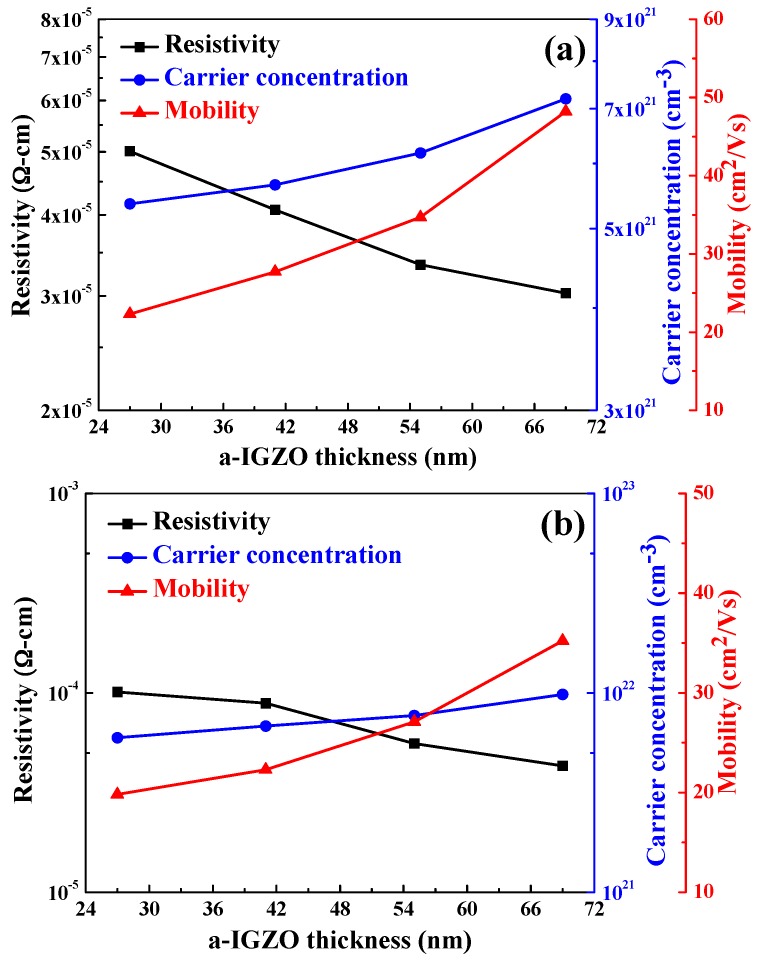
Resistivity, Hall mobility, and carrier concentration of the α-IGZO/Ag/α-IGZO triple-layer structure as a function of thickness of IGZO film. On (**a**) glass substrates; and (**b**) PET substrates.

Figure 9 exhibits the resistivity, carrier concentration, and mobility of the α-IGZO/Ag/α-IGZO triple-layer structure as a function of thickness of IGZO films. As Figure 9a shows, as glass was used as the substrate, the carrier concentration increased from 5.36 × 10^21^ cm^−3^ to 7.21 × 10^21^ cm^−3^ and the mobility increased from 22.3 cm^2^/V to 48.2 cm^2^/V as thickness of IGZO films increased from 27 nm to 69 nm. The resistivity of the α-IGZO/Ag/α-IGZO triple-layer structure slightly decreased from 5.01 × 10^−5^ to 3.03 × 10^−5^ Ω-cm as the thickness of α-IGZO films increased from 27 nm to 69 nm. As Figure 9b shows, as flexible PET was used as the substrate, the carrier concentration increased from 5.97 × 10^21^ cm^−3^ to 9.81 × 10^21^ cm^−3^ and the mobility increased from 19.8 cm^2^/V to 35.3 cm^2^/V as thickness of IGZO films deposited on PET substrates increased from 27 nm to 69 nm. The resistivity of the α-IGZO/Ag/α-IGZO triple-layer structure slightly decreased from 1.12 × 10^−4^ to 4.31 × 10^−5^ Ω-cm as the thickness of α-IGZO films deposited on PET substrates increased from 27 nm to 69 nm. These results in Figure 9 show that the carrier concentration critically increased and the mobility slightly increased as the thickness of IGZO films increased, independent of the substrate used. The decrease in resistivity is mainly due to the increase of carrier concentration and carrier mobility. In addition, Figure 9 shows that the resistance of the α-IGZO/Ag/α-IGZO triple-layer structure has no large variation as the thickness of α-IGZO films increased. The small variation in resistance could be attributed to the thickness of Ag layers being identical in all α-IGZO/Ag/α-IGZO triple-layer structures.

**Figure 10:**
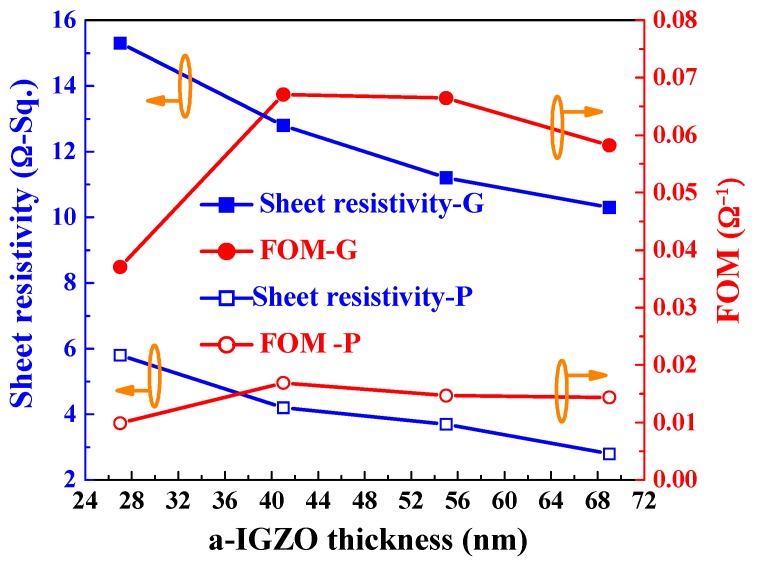
FOM values of the α-IGZO/Ag/α-IGZO triple-layer structure as a function of thickness of α-IGZO film.

**Figure 11:**
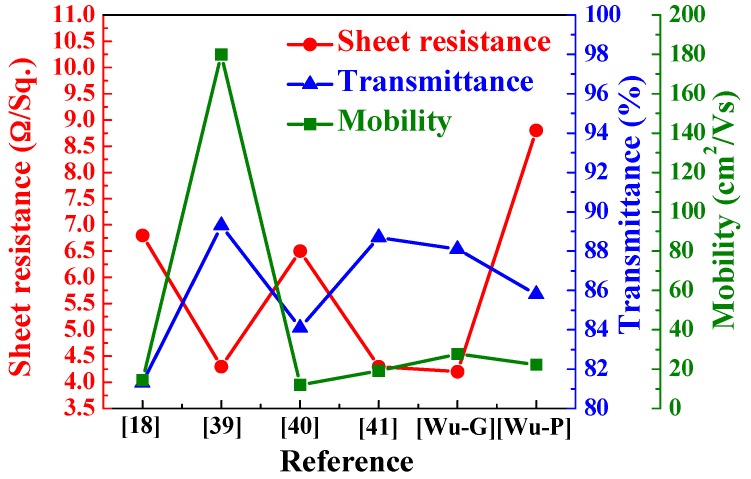
Performances of the α-IGZO/Ag/α-IGZO films as compared with the data in the references [18,39,40,41]. [Wu]: the data investigated in this study.

The FOM value was also calculated using the Equations (4) and (5) with an Ag film thickness of 13 nm and as a function of thickness of α-IGZO films, and the calculated results are shown in Figure 10. As the Figure 10 shows, as the thickness of α-IGZO films increased, the FOM value first increased, reaching a maximum at 41 nm, and then decreased as the α-IGZO thickness further increased. As the thickness of α-IGZO was 41 nm and glass was used as the substrate, the α-IGZO/Ag/α-IGZO triple-layer structure shows the properties of maximum FOM value of 0.067 Ω^−1^, sheet resistance of 4.2 Ω/□, and average optical transmittance (350 nm~650 nm) of 88.1%, respectively. As the thickness of α-IGZO was 41 nm, the α-IGZO/Ag/α-IGZO triple-layer structure deposition on PET substrate shows the properties of maximum FOM value of 0.017 Ω^−1^, sheet resistance of 12.8 Ω/□, and average optical transmittance (350 nm–650 nm) of 85.8%, respectively. The optimal sheet resistance, optical transmittance ratio, and carrier mobility of the α-IGZO/Ag/α-IGZO triple-layer structure deposited on glass substrate is better than and on PET substrate, having comparable results with the data in other studies, for example ITO/Ag/ITO, ITO/Ag-Pd-Cu/ITO, IGZO/Ag/IGZO, and other triple-layer structures [18,39,40,41], as shown in Figure 11. In this study, the high optical transmittance and good electrical conductivity of the α-IGZO/Ag/α-IGZO triple-layer structure can be obtained as the Ag and α-IGZO thickness are set to 13 and 41 nm, respectively.

## 4. Conclusions

The optical and electrical properties of the triple-layer structure transparent conductive oxide thin films, α-IGZO/Ag/α-IGZO, were well developed in this study. As the thickness of the top and bottom α-IGZO films was controlled at 40 nm and thickness of Ag film increased from 10 nm to 16 nm, the carrier concentration increased from 4.09 × 10^21^ cm^−3^ to 5.99 × 10^21^ cm^−3^, the mobility increased from 11 cm^2^/V to 36 cm^2^/V, and the resistivity decreased from 5.99 × 10^−5^ to 2.39 × 10^−5^ Ω-cm, respectively. As the thickness of Ag film was set at 13 nm and the thickness of IGZO films increased from 27 nm to 69 nm in the top and bottom layers, as glass (PET) was used as the substrate, the carrier concentration increased from 5.36 × 10^21^ cm^−3^ to 7.21 × 10^21^ cm^−3^ (from 5.97 × 10^21^ cm^−3^ to 9.81 × 10^21^ cm^−3^), the mobility increased from 22.3 cm^2^/V to 48.2 cm^2^/V (19.8 cm^2^/V to 35.3 cm^2^/V), and the resistivity slightly decreased from 5.01 × 10^−5^ to 3.03 × 10^−5^ Ω-cm (1.12 × 10^−4^ to 4.31 × 10^−5^ Ω-cm), respectively. As the α-IGZO and Ag films had the thicknesses of 40 nm and 13 nm, as glass (PET) was used as the substrate, the α-IGZO/Ag/α-IGZO triple-layer structure had the maximum FOM of 0.067 Ω^−1^ (0.017 Ω^−1^), small sheet resistance 4.2 Ω/□ (12.8 Ω/□), and high average optical transmittance ratio of 88.1%, respectively. Those results indicate that α-IGZO/Ag/α-IGZO triple-layer structures are a promising low-cost, low toxicity, low-temperature processing material for touch panel application and for future flexible applications.
